# Sustainable activated carbon from palm waste for aqueous nickel II adsorption

**DOI:** 10.1038/s41598-026-37088-8

**Published:** 2026-02-14

**Authors:** W. A. Hammad, Mohamed S. Abdel-latif, Samah A. Hawash, Mohammed Kuku, M. H. A. Amr

**Affiliations:** 1 Faculty of Engineering, Tanta University, Tanta, Egypt; 2 Department of Chemical Engineering, Menoufia Higher Institute of Engineering and Technology (MNF-HIET), Menoufia, Egypt; 3 Department of Mechanical Engineering, College of Engineering and Computer Science, Jazan University, Jazan, Saudi Arabia; 4 Engineering and Technology Research Center, Jazan University, P.O. Box 114, Jazan, 82817 Saudi Arabia; 5 Tanta Higher Institute of Engineering and Technology, Tanta, Egypt

**Keywords:** Adsorption, Activated carbon, Heavy metals, Nickel(II), Palm fronds, Contamination, Wastewater treatment, Environmental sciences, Chemistry, Engineering, Materials science, Physics

## Abstract

Industrial wastewater discharge remains a major environmental concern, with heavy metal contamination posing significant risks to ecosystems and public health. Among these, Ni (II) is commonly detected in effluents and is particularly hazardous when present in drinking water. Adsorption has gained attention as a simple, cost-effective, and efficient method for removing such contaminants.This study investigates the use of AC derived from palm fronds and treated with H₃PO_4_ (PFTAC) for the adsorption of Ni(II) ions using batch experiment tests. The material demonstrated outstanding performance, achieving a 99.65% removal efficiency within 90 min at an initial concentration of 50 ppm under neutral pH conditions. Nitrogen sorption analysis revealed the mesoporous structure of PFTAC, supported by characteristic adsorption-desorption isotherms. Surface area and pore characteristics were evaluated using BET, t-plot, and BJH methods. Comprehensive material characterization was conducted using SEM (surface morphology), FT-IR (functional groups), and XRD (crystalline structure). These analyses confirmed the suitability of PFTAC as a high-performance, sustainable adsorbent for Ni (II) removal from industrial wastewater. Adsorption onto PFTAC followed pseudo-second-order kinetics (R² = 0.9957), indicating chemisorption. Thermodynamic results confirmed a spontaneous, endothermic process. Langmuir isotherm best describes the equilibrium (R² = 0.9998) with a maximum capacity of 166.7 mg/g.Ni(II) removal using PFTAC was optimized using Box-Behnken Design. The model showed high accuracy (R² = 0.991) and significance (*p* < 0.05). ANOVA and residual analysis confirmed model reliability. Key factors were time, concentration, and temperature.

## Introduction

People worldwide desire and need access to clean water as a basic human right^1^. the world is experiencing a water shortage due to increased pollution as the limited freshwater supplies are depleted. Due to inappropriate waste disposal, there has been a decline in the number of water resources accessible while water consumption has increased at a geometric pace. It is crucial to highlight that the rate at which human tissues absorb these heavy metals outpaces the rate at which they are eliminated through destructive metabolism^2^.

There are numerous environmental factors, including human activity, that contribute to water contamination, include There are numerous environmental factors, including human activity, that contribute to water contamination: industrial waste residues, sewage waste, chemical pesticides, agricultural chemical fertilizers, agricultural wastewater, radioactive pollutants, algae, and oil and its derivatives.

The most dangerous source of pollution is industrial waste residues, where industrial waste is one of the main contributors to the contamination of rivers, seas, and oceans, and these wastes discharge numerous harmful compounds into water bodies like rivers, seas, agricultural drains, or sewage.

The different types of chemicals in wastewater depend on the type of existing industries and the type of treatment in each plant. Most factories throw a lot of substances such as acids, bases, industrial detergents, dyes, some phosphorus compounds, and harmful heavy metals, including Ni(II), lead, and mercury, which cause severe pollution of the water in which they are thrown^3^.

Industrialized countries dispose of solid waste by burying it in the ground at different depths. As for liquid waste, it is thrown into surface pools of varying depths. Rainfall and the rise in the groundwater level lead to the dissolution of some of these wastes and their seepage into the groundwater^4^. –^5^.

A class of compounds known as heavy metals comprises metals and metalloids with atomic weights ranging from roughly 64 to 200^6^. heavy metal elements naturally appeared in its crust when the Earth was forming. However, anthropogenic activities like metal mining, chemical fertilizers, and industrial manufacturing have caused an impending surge in metallic substances in terrestrial and marine environments^7^.

Because heavy metal water pollution harms the ecosystem and human health, One of the most crucial environmental challenges is this. Heavy metals can be found in untreated or partially treated effluents from mining, electroplating, leather tanning, water cooling, and pigment manufacturing^8^.

Surface water and soil can become naturally polluted by (Ni) (II), which occurs naturally in significant quantities in the earth’s crust and core^7^.

The industries involved in electroplating, metal surface treatment, conversion coating, anodizing cleaning, milling, etching, electrolysis depositions, petroleum refining, and battery manufacturing are the sources of Ni(II) production^9^.

Even though Ni(II) is a vital biological component for the healthy development of many animals, excessive levels can have hazardous side effects, including respiratory problems, kidney inflammation, and significant overall weakness. Ni (II)has also raised concerns as a potential carcinogen^7^.

The human body is also subject to acute and long-term impacts from Ni(II) ions. It harms infants’ immune systems, respiratory systems, reproductive systems, hearts, and fetal development^9^.

Ni(II)is a human carcinogen. Chronic exposure to Ni(II) is a risk factor for lung cancer. Nickel(II) is the most common allergenic metal^10^.

Ni(II) was chosen among various heavy metals because it is both an essential trace element and a toxic pollutant widely released from electroplating, alloy production, and battery industries. Its high solubility and mobility increase environmental persistence and bioaccumulation risks. Moreover, Ni(II) is a common allergen and a recognized human carcinogen, particularly linked to respiratory diseases and lung cancer, making it a priority pollutant for removal studies as mention above in details.

To offer a lot of fresh water for irrigation, it is important to reuse wastewater to irrigate agricultural regions once it has been purified. Water purification techniques must also be effective, affordable, long-lasting, and often reused^11^.

**Fig. 1:**
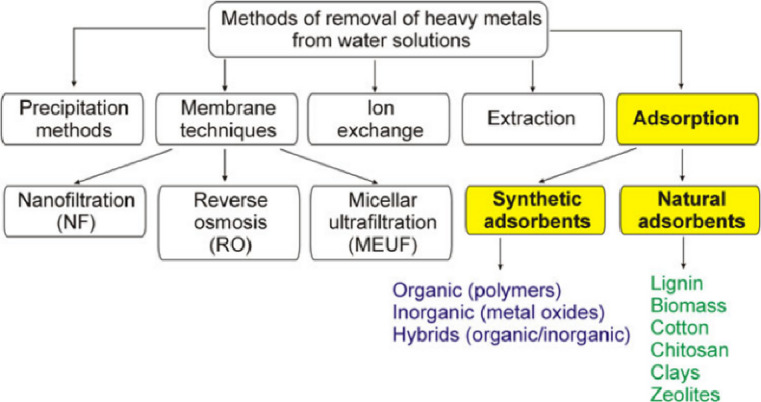
Techniques for eliminating heavy metals from wastewater^13^.

Chemical coagulation, oxidation, coagulation, coagulation, ozone, membrane/nanofiltration, radiation, ion exchange, and biological treatment technologies are just a few techniques to remove heavy metal ions from wastewater Fig. 1. However, most of these methods have flaws, including high sludge formation and low removal efficiency, substantial capital, operating, and technological requirements. As a result, these techniques do not apply to tiny industries in underdeveloped nations. Adopting low-cost wastewater treatment technologies that will maximize the use of scarce water resources and guarantee compliance with all health and safety regulations for reusing treated wastewater is the main difficulty in implementing this approach^12^.

Because it is so effective at removing heavy metals, adsorption is a viable alternative therapy. Due to their high adsorption capacity, common adsorbents such as activated carbon, zeolite, and clay are widely used. According to studies, once considered harmful to the environment, agricultural wastes can now be used to detoxify industrial metal-bearing effluents and remove heavy metals. Agricultural trash includes empty fruit bunches from oil palm trees, bean husks, raw pine cones, durian tree sawdust, coconut coir, rice straw, pomelo peel, and palm fronds^14^.

Natural adsorbents are abundant and easily accessible materials. They have various traits, including high surface area and a significant capacity for cation exchange, both of which are essential components of conventional adsorbents. Additionally, they are substantially less expensive than conventional al adsorbents in terms of price.

A practical, widely available, and sustainable alternative for wastewater applications is agricultural waste products. Agricultural wastes may easily be converted into activated carbon, which has a better chance of adsorbing heavy metals because they are often dense and have low ash content^3^.

As previously mentioned, using adsorption techniques to reduce the amount of contaminants entering water bodies is a frequent practice. Researchers strive to produce activated carbons from inexpensive sources to replace expensive commercially activated ones. Holes with a low volume broaden the surface area for chemical or adsorption reactions differentiating biomass-based activated carbons. Significant microporosity, the surface area that varied based on the type of raw material, and the carbonization process were other characteristics of activated carbon^15^.

**Fig. 2:**
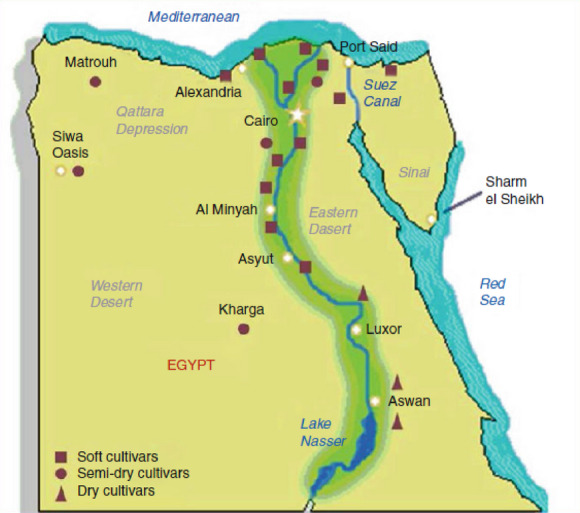
Egyptian date palm cultivars’ geographic distribution^16^.

Egypt has a very long history of date palm cultivation. The date palm tree is very useful nutritionally and economically in Egypt. It is a significant fruit tree and the ideal crop to be grown because of its historical uses as a key food source and byproducts and its ecological advantages in oasis agriculture Fig. 2. The world’s highest date palm fruit production is found in Egypt. There is a great opportunity to expand the date palm production area to meet the national need and produce date fruits for export.

Many goods and services, including many basics of life, are produced by date palms. The fruit of the date palm, which is high in protein, vitamins, and mineral salts, is its main product. Due to the annual pruning, the cultivator can use the palm’s secondary products.

When each palm generates more than 25 kg annually, and there are more than 17 million palm trees in Egypt, palm leaves are regarded as a significant field residue produced annually in Egypt and the Arab countries. Instead of burning them, these field wastes can be employed in many sectors or to extract valuable natural resources because they are rich in cellulose, carbohydrates, and other vital components.

**Fig. 3:**
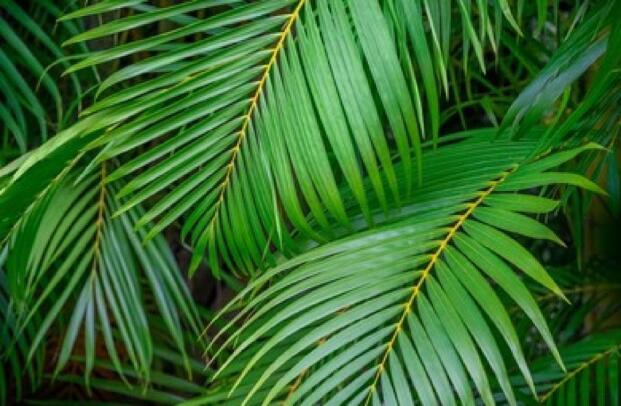
Palm frond^18^.

Palm frond (Fig. 3)fiber is an agricultural waste that is no longer used. In addition, the palm is a sustainable source of palm fibers, which is a very good starting material as it is strong and, at the same time, not thick so that it is converted into dehydrated carbon efficiently, easily, and with low costs, with a high yield of up to 90%^17^.

Although several studies have investigated the adsorption of Ni(II) using various activated carbons, limited research has focused on the use of phosphoric acid–treated palm frond carbon and its modeling performance. Therefore, this study aims to fill this gap by evaluating the adsorption efficiency and developing a predictive model for Ni(II) removal using this novel biosorbent.

This research sheds light on the possibility of using palm fronds to purify water, especially industrial wastewater, and rid it of heavy elements such as Ni (II)before it reaches the public sewage system.

The palm frond samples used in this study were collected from Egyptian palm fields located in Rashid (Rosetta), Egypt.

## Materials and methods

### Adsorbent material preparation

Samples of palm fronds were divided into 1 to 3 cm pieces. Each piece was carefully cleaned in tap water to remove the dust, distilled fibers, and other pollutants. To reduce the moisture content, samples of washed palm fronds were dried at 105 °C for 5 h in an oven (Lenton). The powdered dried palm fronds (RDFs) were processed. First, a 60% solution of H_3_PO_4_ was used to impregnate the powder to create activated carbon (ACs). Of samples, 5 g Weighed palm fronds were combined with 15 cc of 60% H_3_PO_4_. The samples were then impregnated in a muffle furnace for two hours at 100 °C, followed by three hours of activation at 400 °C. The sample was washed to remove the acid content of the manufactured activated carbon. Up till pH 7 was reached, the washing procedure was continued. After that, the samples were dried in an oven at 110 °C to remove any moisture^19^.

### Preparation of nickel(II) stock solution

A stock solution of Ni(II)concentration 1000 mg/l was prepared by dissolving 4 g of Ni(II) Sulfate into 1000 ml of distilled water. Dilute Ni(II) ions stock solution from 1000 mg/l was diluted to different concentrations using distilled water under dilution law. All chemicals used were of analytical grade with 99.9% assay. The pH of solutions was adjusted to the required value by adding 0.1 N NaOH or 0.1 N HCl at a fixed temperature of 25 °C^20^.

### Adsorption experiment

**Fig. 4:**
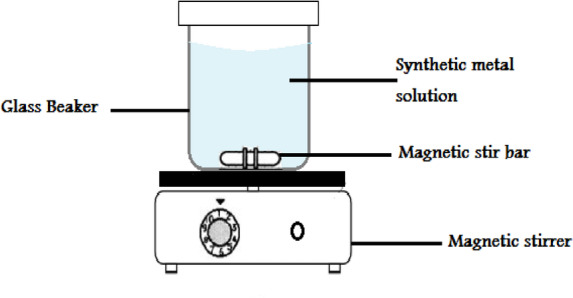
Schematic diagram of the apparatus used for the adsorption process.

**Fig. 5:**
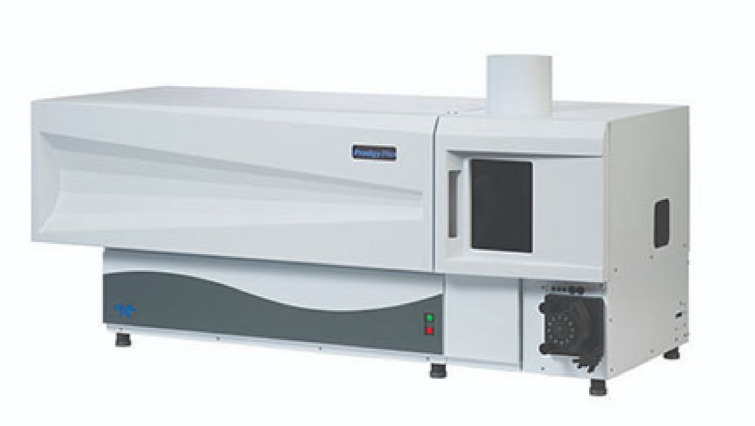
Inductively coupled plasma-optical emission spectrometry.

Ni(II) adsorption studies onto activated carbon prepared from palm fronds treated with H_3_PO_4_ were performed in 250 ml of Ni(II) by batch equilibrium method. The following process variables were employed for the adsorption experiments: Temperatures of 20–45 °C, pH 2–8, starting metal concentrations of 50–300 mg/l, and contact times of 5–90 min. The initial response With 0.1 M HCl or NaOH solution, the pH was changed. A desired quantity of adsorbents was introduced into a beaker containing a Ni (II) solution to establish equilibrium. The solution was then stirred at 3000 rpm using a ceramic hot plate stirrer (Model C230V50/60Hz) at the desired contact time. Figure 4 A test sample of 5 ml of solution was obtained after the appropriate amount of time had passed, and it was centrifuged (Model C230V50/60Hz) for 15 min at 4000 rpm to separate the adsorbents from the liquid phase. The supernatant was preserved for metal concentration measurements using the Prodigy Plus ICP-OES inductively coupled plasma-optical emission spectrometry Fig. 5.

The percentage of adsorption (%) is calculated using the following Eq. (1)

1 $$\:\mathrm{\%}\:Removal=\left(\frac{{C}_{0}-{C}_{t}}{{C}_{0}}\right)\times\:100$$

C_0_ and C_t_ are the initial concentration and equilibrium concentration of Ni(II) in the solution at (t) time (ppm), respectively^21^.

The surface area and porosity of the prepared sample were determined from N_2_ adsorption measured at −176 °C using BELSORP max II equipment, Japan. The sample was initially out-gassed under vacuum conditions for 24 h (10^− 4^ Torr) at 100 °C for on. When all of the pores in the sample are filled with liquid due to saturation of the pressure, the fine pore structure of the sample can be assessed. After then, the adsorptive gas pressure gradually decreased, evaporating the condensed gas from the system. The information was utilized to create a relationship between adsorption and desorption isotherms. Using the adsorption isotherm, the amount adsorbed is displayed as a function of pressure at a constant temperature. The Brunauer-Emmett-Teller (BET) method calculates the specific surface area^22^. According to the t-plot approach, the exterior surface area, micropore volume, and total pore volume were calculated. Using the Barnett-Joyner-Halenda (BJH) technique, the microporous volume and pore size distribution were estimated.

To analyze the surface morphology of the samples, scanning electron microscopy with energy-dispersive X-ray analysis (SEM-EDX) was evaluated as a supplemental technique to wet chemical analysis. A VEGA II LMU (Tescan) scanning electron microscope outfitted with INCA Energy 450/XT (Silicon Drift detector, SDD) and INCA Wave 700 (crystals: LiF, PET, TAP, LSM60, LSM200) EDS equipment was used to evaluate the micro-texture and elemental composition of the samples. The INCA Energy Plus software suite enables simultaneous use of both systems. The BSE detector was used to obtain the SEM images. Perkin Elmer Spectrum 2 picked up the FT-IR spectrum. PW3040/60 Panalytical Diffractometer was used to investigate the powder’s XRD pattern.

## Results and discussion

### Characterization of solid adsorbate

**Fig. 6:**
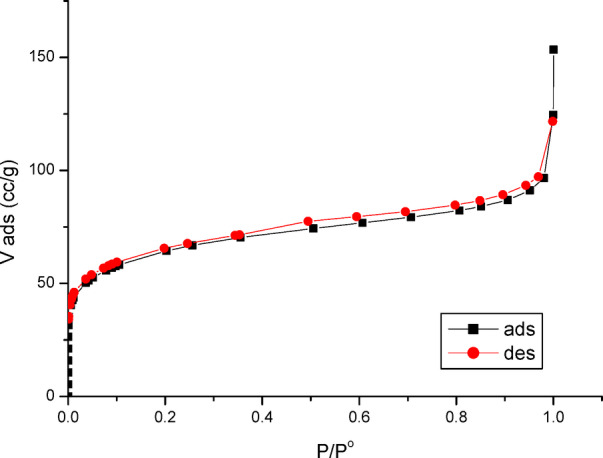
Nitrogen adsorption-desorption isotherm before the adsorption processes.

The nitrogen adsorption-desorption isotherm plot PFTAC before the adsorption processes is illustrated in Figure 6.

PFTAC isotherm is of Type IIb, exhibiting an H4 hysteresis loop at high relative pressure. It is worth mentioning here that Type IIb is a new classification proposed by F. for Type IV-like isotherms but without a plateau at high P/P°^23^. A frequent explanation for the forming hysteresis loop in the P/P° range of 0.4 to 0.9 is capillary condensation in the mesopores^24,25^. Types H4 hysteresis, frequently observed in activated carbon, confirm the presence of slit-shaped holes, mainly in the micropore range.

**Fig. 7:**
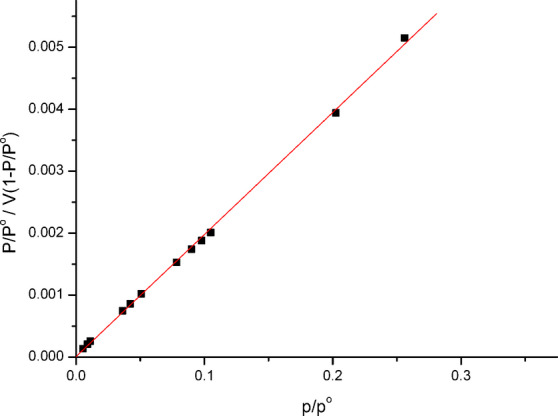
BET surface area plot before adsorption.

The most appropriate correlation for the equilibrium curves should be constructed to optimize the adsorption system. Figure 7 depicts a linearized model of nitrogen multilayer adsorption as determined by the Brunauer-Emmet-Teller (BET) method as a function of relative pressure.

To calculate the overall specific surface area in m^2^/g, evaluations of the exterior area and pore area are included. The following is a typical way to write the BET equation:

2 $$\:\frac{P/{P}^{o}}{V[1-P/{P}^{o}]}\:=\:\frac{c-1}{{V}_{m}}\:\left(\frac{P}{{P}^{o}}\right)+\:\frac{1}{{V}_{m\:}C}$$

where P and P are the equilibrium and the saturation pressure of adsorbate at the temperature of adsorption, respectively; *V* is the adsorbed gas quantity while *V*m is the monolayer adsorbed gas quantity, and C is the BET constant Fig. 6.

According to experimental findings, Eq. (2) is an adsorption isotherm and may be shown as a straight line with 1/V(1-P/P^o^) on the Y-axis and P/P^o^ on the X-axis. A BET storyline is what this one is. This equation’s linear relationship is only maintained within the range of 0.05 P/P^o^ 0.35. The monolayer adsorbed gas quantity V_m_ and the BET constant C are calculated using the line’s slope (C-1/V_m_C) and intercept (1/V_m_C) values. The mean pore radius r, total pore volume V_T_, and specific surface area S_BET_ were computed. The following summarizes the findings *V*_*m*_=80.7 cc/g; *C* = 1.002; S_BET_=352.66 m^2^/g; V_T_=91.415 cc/g; *r* = 8.02 nm. It is important to note that date stone-based activated carbon with a greater surface area was produced by activating carbon with H_3_PO_4_ acid^26^.

The Pore Size Distributions were calculated using the Barret, Joyner, and Halenda (BJH) pore size model, PSD, spanning a portion of the micropore range. And the mesopore range BJH pore size

3 $$\:{r}_{p}={r}_{k}+t$$

Where r_p_ is the pore’s actual radius r_K_ is the pore’s Kelvin radius, and **t** = thickness of the adsorbed film.

**Fig. 8:**
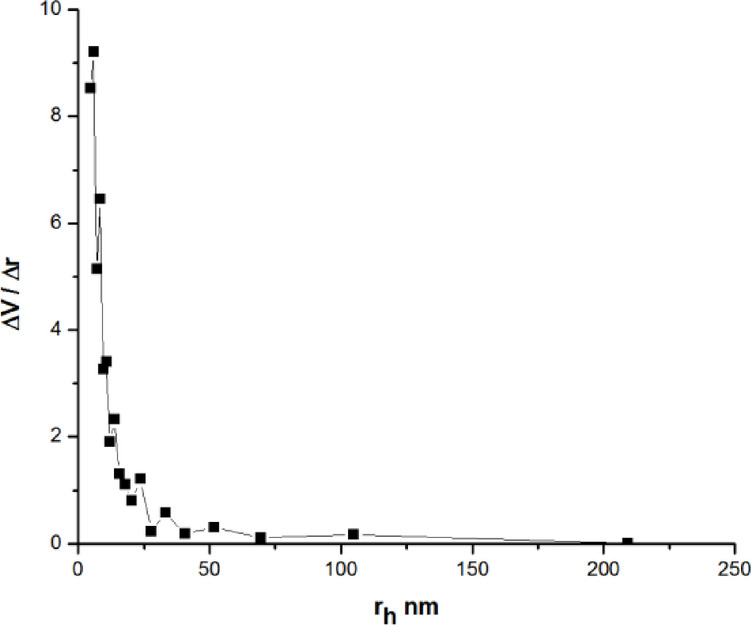
Pore size distribution by BJH before adsorption.

BJH accounts for the statistically significant drop in pore thickness throughout the desorption process. Figure 8 displays the sample’s pore size distributions, which were determined using the BJH method from the desorption branches of isotherms. It represents the mesopore region’s distribution of pore sizes.

**Fig. 9:**
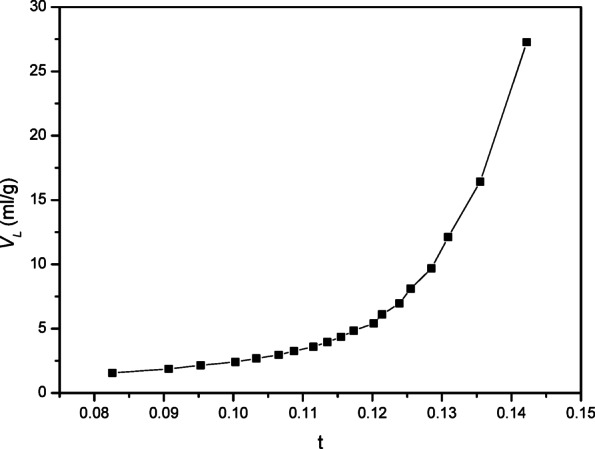
T-plots original samples.

Using the t-plot approach, the experimental data were processed to produce exterior surface area, micropore volume, and total pore volume. A mathematical model of the multilayer was used to determine layer thickness, or “t,” as a function of rising p/po. The adsorbate layer’s thickness, “t,” increases as pressure increases. Figure 9plots the adsorbed (VL) experimental volume versus the statistical thickness (t) for each p/po. The sample exhibits an upward deviation, indicating mesoporosity, according to Lippens and de Boer’s classification. The slope of the straight lines through the origin was used to compute the surface area St(m2/g), and these values match the SBET value exactly^27^.

The results summarized in Table 1.

**Table 1:** Surface area, pore volume, and pore size characteristics of PFTAC before adsorption.

Sample	SBET (m²/g)	Total Pore Volume, VT (cc/g)	Average Pore Radius, *r* (nm)	Vm (cc/g)	BET Constant, C	Notes
PFTAC (before adsorption)	352.66	91.415	8.02	80.7	1.002	Type IIb isotherm, H4 hysteresis loop, mesoporous with slit-shaped micropores

**Fig. 10:**
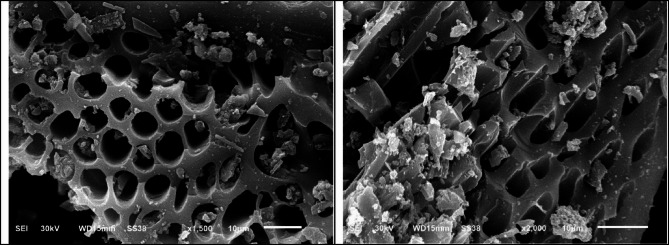
Scanning electron micrograph (SEM) of PFTAC.

The surface morphology studied by SEM Fig. 10; shows that the PFTAC and Ni(II)adsorbed samples on PFTAC were porous. The surface’s topography matches the surface area value and porosity from BET, t-plot, and BJH measurements. Results of the SEM–EDX analysis Figs, 11, 12 of the sample adsorbed Ni(II)confirmed the presence of Ni(II)with a concentration of 2 wt%.

**Fig. 11:**
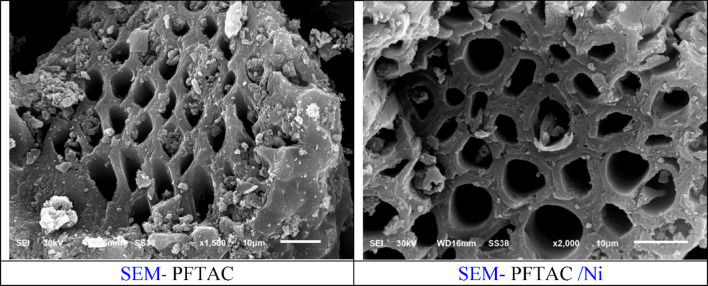
Scanning electron micrograph (SEM) of Ni(II)adsorbed on PFTAC.

**Fig. 12:**
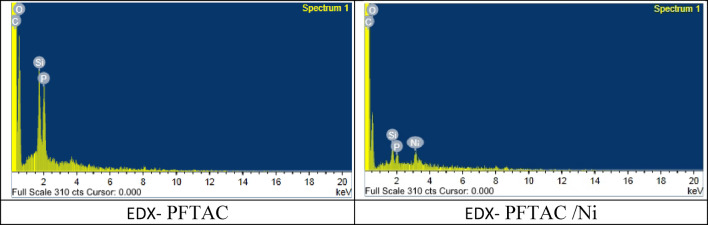
EDX of PFTAC and Ni(II)adsorbed on PFTAC (PFTAC/Ni).

It is noteworthy that the elemental composition of the PFTAC surface, as determined by mapping, revealed a high concentration of silica and phosphorous, two alkaline earth metals.This characteristic enhances the possibility of removing metals using precipitation or ion exchange processes.

**Fig. 13:**
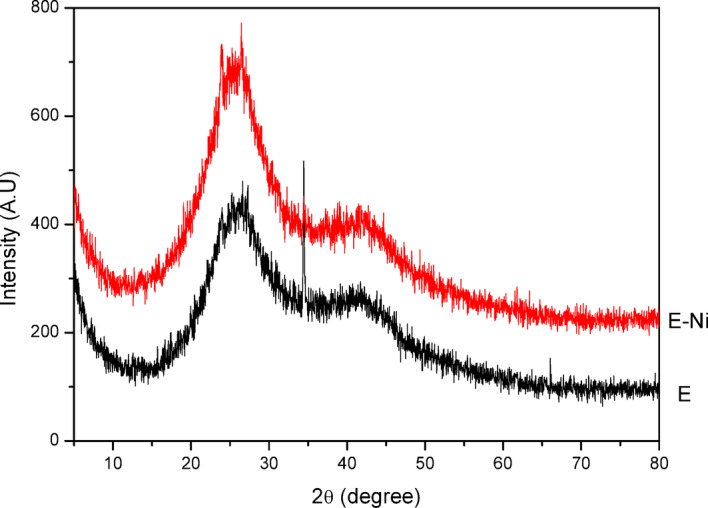
XRD pattern of PFTAC and Ni(II) adsorbed on PFTAC (PFTAC/Ni).

Figure 13 shows the XRD pattern recorded for PFTAC and Ni(II)adsorbed on PFTAC (PFTAC/Ni) samples using CuKα (λ = 1.54 Å) as a source of radiation with a 2θ scanning range from 5 to 80. The diffraction pattern shows a broad peak at 2θ = 26, which indicates the amorphous nature of PFTAC, and Ni(II)dsorbed on PFTAC (PFTAC/Ni). Further, the recorded XRD pattern reveals no ordered crystalline structure^28^.

**Fig. 14:**
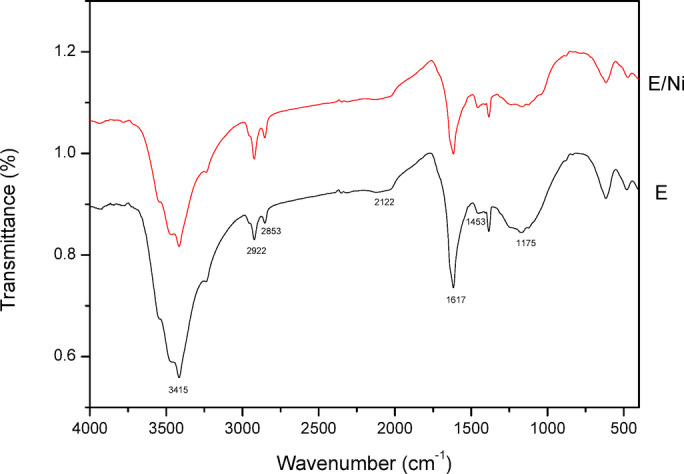
FT‒IR spectra of PFTAC and Ni(II)adsorbed on PFTAC (PFTAC/Ni).

Figure 14 displays the FT-IR (KBr, cm^− 1^) of PFTAC at the following wavelengths: 3415 cm^− 1^ (OH), 2922 and 2853 cm^− 1^ (CH-from.), 2122 cm^− 1^ (CH-aliphatic.), 1617 cm^− 1^ (C = O stretching of acid), 1453 cm^− 1^ (C = C stretch), 1175 cm^− 1^ (C-H bending vibration). FTIR spectral data indicate the presence of flavonoids and polyphenols in PFTAC. The FT-IR of PFTAC and Ni(II)adsorbed on PFTAC (PFTAC/Ni) did not change. That means the adsorption is physical^28^.

The uptake mechanism of Ni(II) onto the H₃PO₄-treated palm frond activated carbon (ACTPF) primarily involves surface complexation, electrostatic attraction, and ion exchange between Ni(II)ions and oxygenated functional groups such as hydroxyl, carboxyl, and phosphate moieties. The porous structure and high surface area of ACTPF facilitate both physical and chemical adsorption processes. Furthermore, the reusability of the adsorbent is crucial for practical wastewater treatment applications. The regenerated ACTPF showed stable adsorption efficiency over multiple cycles, indicating its potential for real industrial wastewater utilization and cost-effective environmental remediation.

### Effect of pH

**Fig. 15:**
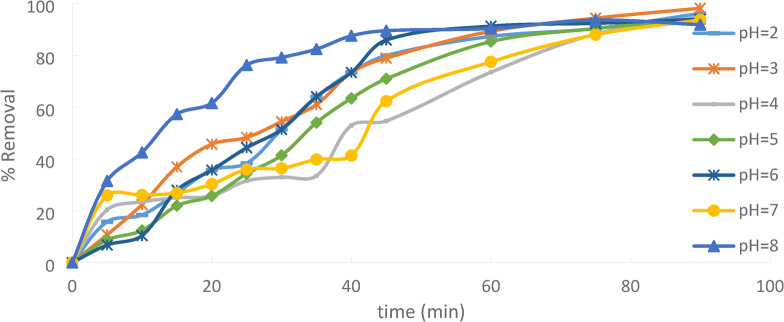
pH effect regarding Ni(II) adsorption onto PFTAC (initial concentration = 50 ppm, PFTAC dose = 0.5 g/250 ml, stirring speed = 300 rpm T = 25 ℃ and time = 90 min).

To extract Ni(II) from aqueous solutions, pH is a crucial adsorption parameter. It affects the surface charges, ionization level, and adsorbate specifications. To attain perfect equilibrium, the initial Ni(II) concentration must be 50 ppm, and the contact period for adsorption must be 90 min. To comprehend the impact of pH on Ni(II) adsorption, equilibrium studies at various pH values of 2 to 8 are used. ROME, HANNA pH 211 The pH of solutions was adjusted using a pH meter Fig. 15.

Electrostatic interactions, solubility, and the general adsorption process are all impacted by pH, which also impacts the ionization states of the adsorbent and the adsorbate. Acidic pH: May protonate the adsorbate and adsorbent surface, which, depending on their respective properties, may help or impede adsorption.Basic pH: Has the ability to deprotonate the adsorbate and adsorbent surface, which could help or impede adsorption. The ideal pH is where the greatest amount of adsorption takes place; this is usually ascertained via experimentation.

Increasing in pH will increase the % removal till pH = 3^29^, then Increasing in pH will decrease the % removal. Because hydrogen and Ni(II) ions compete for sorption sites at lower pH levels, Ni(II) removal is impeded. Beyond pH = 3, there is a decrease in the amount of adsorbed material due to the precipitation of Nickel hydroxide (Ni(OH)_2_).

**Fig. 16:**
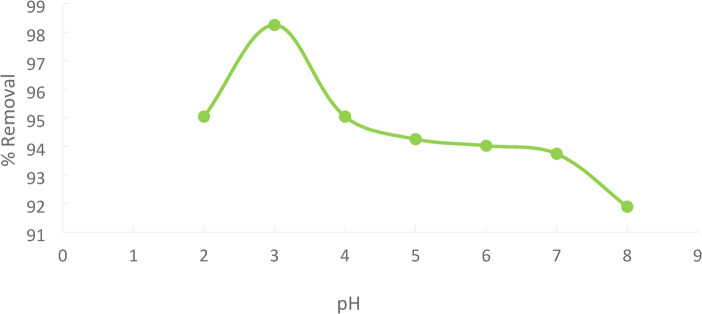
pH effect regarding Ni(II) adsorption.

As the pH rose from 2 to 3, the proportion of Ni(II)removed increased, rising from 96.25% to 98.25%. The greatest removal for Ni(II) was determined to be 98.25% at pH = 3, with % removal and adsorption capacity decreasing with rising pH for pH above 3. Figures 16^30,31^.

### Effect of initial concentration

**Fig. 17:**
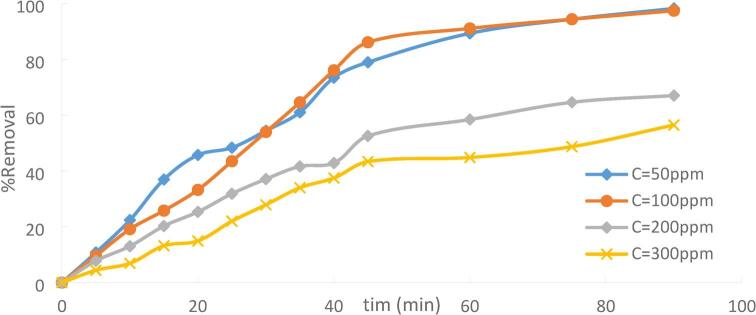
Effect Ni (II)initial concentration adsorption onto PFTAC (pH = 3, PFTAC dose = 0.5 g/250 ml, stirring speed = 300 rpm T = 25℃ and time = 90 min).

The effect of initial Ni(II) concentrations on the percentage removal of Ni(II) onto PFTAC was carried out at contact time = 90 min, pH = 3, stirring speed = 300 rpm, and at a temperature of 25^˚^C. adsorbent dose = 0.5 g/250 ml for different Ni (II)initial concentration (50,100,200 and 300 ppm),. Figures 17demonstrate that as the original Ni(II) concentration has increased, the percentage of Ni(II) removed has reduced. The increasing ratio of the initial number of moles of Ni(II) to the available unoccupied sites can be used to explain the low percentage clearance at higher metal concentrations^32,33^. Ni(II) ions could easily occupy the adsorbent’s absorption sites at low starting heavy metal ion concentrations. However, most accessible adsorption sites were occupied due to the rise in the initial concentration of heavy metal ions^34^. causing the adsorption capacity to decline. As the initial concentration of Ni(II) ions rises.

The driving force for mass transfer and the total adsorption capacity is influenced by the initial concentration of the metal ions in the solution. High Concentration: Offers a stronger adsorption driving force, which may boost the rate and capacity of adsorption until the adsorbent is saturated.Low Concentration: Because of a reduced driving force, this may result in lower adsorption rates and capacity.Optimal Concentration: Find the range of concentrations that maximizes adsorption efficiency while avoiding premature saturation.

**Fig. 18:**
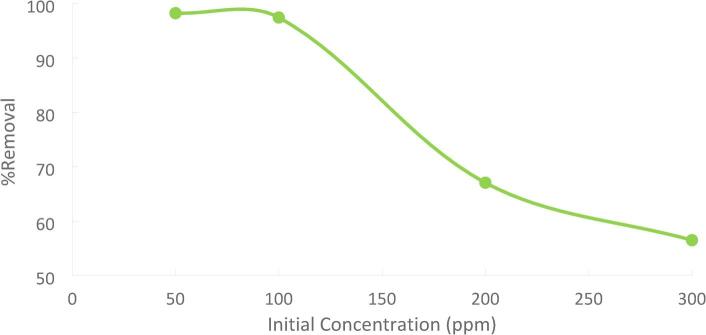
Effect of initial Ni(II) concentration on adsorption.

The result reveals that the percentage removal of Ni(II) decreased from 98.25% to 56.46%, increasing the initial Ni(II) from 50 ppm to 300 ppm. Figures 18.

### Effect of adsorbent dose

**Fig. 19:**
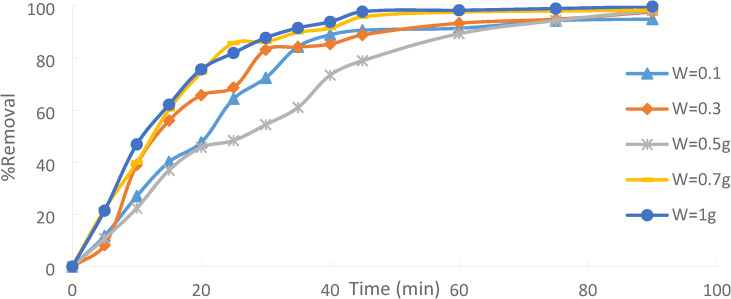
Effect of adsorbent dose of PFTAC on % removal Ni(II) with initial concentration 50 ppm, contact time 90 min, stirring speed = 3000 rpm, 25 ^˚^C and pH = 3.

The experiment was studied at a contact time of 90 min for an initial concentration of 50 ppm Ni(II) at a temperature of 25 °C. Different adsorbent doses (PFTAC) = (0.1, 0.3, 0.5, 0.7, and 1 g/250 ml). Figures 19 demonstrates that as the amount of adsorbent utilized increases, the proportion of Ni(II) removed increases. The adsorption amount depends on the number of open sites above the adsorbent. The adsorbent dosage determines the number of active sites for a particular starting metal ion concentration and is a crucial adsorption parameter. The increased percentage of metal ion elimination with adsorbent dosage may be due to the adsorbent’s surface area increasing the number of adsorption sites available []. 

The capacity and efficiency of adsorption can be affected by the amount of adsorbent used. Greater sample mass can increase the surface area available for adsorption, but it might also make it more difficult to maintain consistent conditions. Small Mass: Because of its constrained surface area, it may have a lesser adsorption capacity.Large Mass: This can increase adsorption capacity but can also result in handling and mixing challenges and saturation consequences. The concept of optimal mass refers to striking a balance between providing a sufficient surface area and avoiding operational difficulties.

**Fig. 20:**
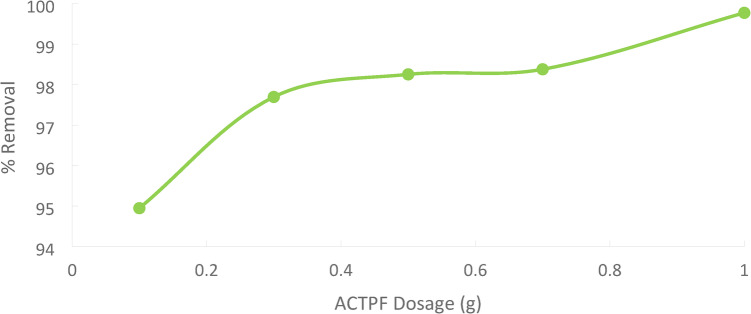
Effect of adsorbent dose % removal Ni(II).

The result reveals that the uptake of % removal of Ni(II) increased from 94.94% to 99.77%, increasing the adsorbent dosage from 0.1 g/250 ml to 1 g/250 ml. Figures 20.

### Effect of temperature

**Fig. 21:**
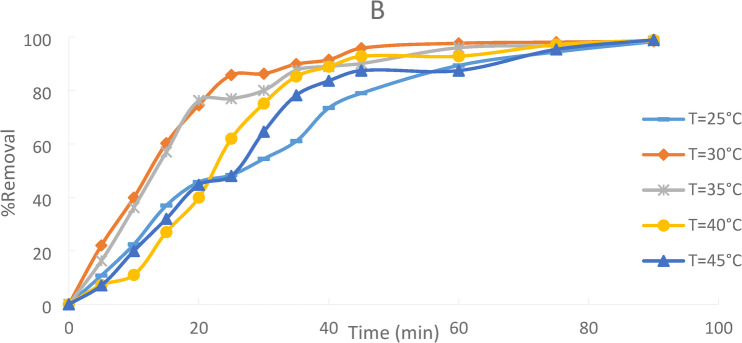
Effect of Temperature on % removal of Ni(II) onto PFTAC, initial Ni(II) concentration 50ppm, adsorbent dose: 1 g\250 ml stirring speed = 300 rpm, contact time 90 min and pH = 3.

Figures 21indicate the effect of temperature on the removal percentage of Ni(II) from wastewater using PFTAC. Different temperatures were used in the study at (25, 30, 35, 40, and 45 °C) with 50 ppm initial Ni(II) concentration in the presence of (1 g/250 ml) for PFTAC. The figure showed that in 90 min, the removal efficiency increased by increasing the temperature. As the temperature rises, the metal ions’ mobilities and mutual retarding forces fall. As a result of better intra-particle Ni(II) ion diffusion into the pores of the adsorbent at the higher temperature, the adsorbent’s capacity, chemical interaction with the adsorbate, and active surface center production are further enhanced. This increase indicates that the adsorption is an endothermic process^38,39^. The promotion of the interaction between the metal ion and the active adsorbent sites, an increase in the mobility of the metal ions, and a decrease in the viscosity of the liquid could all be contributing factors to this improvement in Ni(II) absorption with temperature.

Additionally, higher temperatures will hasten the pace at which Ni (II) diffuses from the solution to the adsorbent’s surface. This is consistent with the adsorption process’ endothermic character^40^. –^41^.

Both the adsorption rate and capacity are impacted by temperature. Temperature variations can either promote or hinder adsorption, depending on whether the process is endothermic or exothermic. High Temperatures: Higher temperatures can potentially improve adsorption capacity if the adsorption is endothermic or heat-absorbing. Conversely, greater temperatures may reduce adsorption capacity if the process is exothermic, emitting heat. Low temperatures are ideal for operations involving exothermic adsorption. The ideal temperature range is one in which the adsorption capacity is maximized without leading to the adsorbent or adsorbate degrading.

**Fig. 22:**
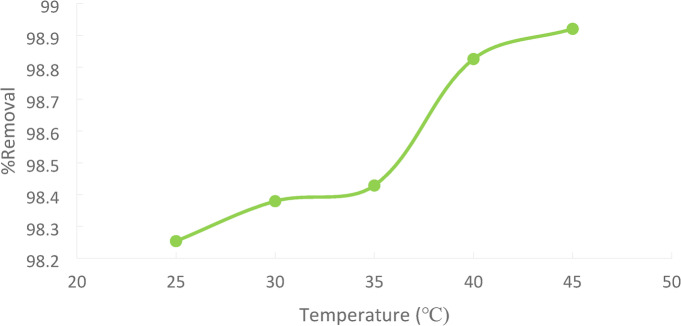
Effect of Temperature on % Removal Ni (II).

The result reveals that uptake of % removal of Ni (II)increased from 98.25% to 99.92% with increasing the temperature from 25℃ to 45℃.figure 22.

### Adsorption of kinetics

#### Pseudo–first order model

The pseudo-first-order equation is most likely the earliest example of a rate of adsorption equation still in use today. Equation 4 produced a pseudo-first-order kinetic model, which was as follows.

4 $$\:\mathrm{L}\mathrm{n}\:\left({\mathrm{q}}_{\mathrm{e}}-{\mathrm{q}}_{\mathrm{t}}\right)=\mathrm{L}\mathrm{n}\left({\mathrm{q}}_{\mathrm{e}}\right)-{\mathrm{k}}_{1}\mathrm{t}$$

the form in Eq. (4) for the boundary condition of t = 0, qt = 0 and t = t

5 $$\:{\mathrm{q}}_{\mathrm{t}}=\frac{\left({\mathrm{C}}_{0}-{\mathrm{C}}_{\mathrm{t}}\right)\times\:\mathrm{v}}{\mathrm{m}}$$

6 $$\:{\mathrm{q}}_{\mathrm{e}}=\frac{({\mathrm{C}}_{0}-{\mathrm{C}}_{\mathrm{e}})\times\:\mathrm{v}}{\mathrm{m}}$$

**Fig. 23:**
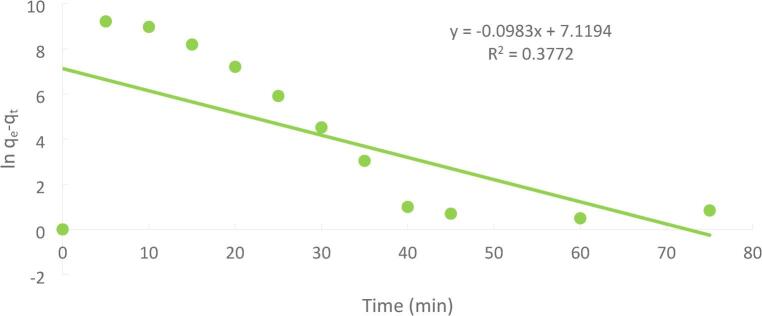
Pseudo-first order kinetic (initial concentration = 50 ppm, PFTAC dose = 0.5 g/250 ml, stirring speed = 300 rpm T = 301 K and time = 90 min, pH = 3).

The slope and intercept of the graph can be used to derive k1 and qe from the plot of ln (q_e_-q_t_) versus t Figure 23 to provide a linear connection.

### Pseudo–second order model

The second-order kinetics may be tested based on the following Eq. (7):

7 $$\:\frac{{dq}_{t}}{dt}={k}_{2}{\left({q}_{e}-{q}_{t}\right)}^{2}$$

Where k_2_ is the pseudo–second–order rate constant of adsorption (mg g-1 min^− 1^).

The linearized–an integral form of the model:

8 $$\:\frac{\mathbf{t}}{{\mathbf{q}}_{\mathbf{t}}}=\frac{1}{{\mathbf{k}}_{2}{\mathbf{q}}_{\mathbf{e}}^{2}}+\frac{1}{{\mathbf{q}}_{\mathbf{e}}}\mathbf{t}$$

The integral form shown in Eq. (8) predicts that the ratio of the time/adsorbed amount of Ni(II) (t/qt) should be a linear function of time.

**Fig. 24:**
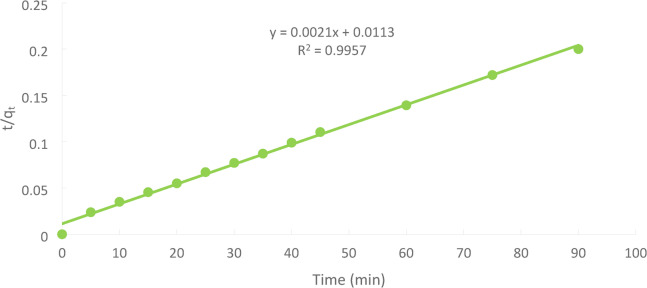
Pseudo-second order kinetic (initial concentration = 50 ppm, PFTAC dose = 0.5 g/250 ml, stirring speed = 300 rpm T = 301 K and time = 90 min, pH = 3).

In our case of Ni(II) adsorption, the relation between the ratio of the time/adsorbed amount of Ni(II) (t/qt) is a linear function of time. (Fig. 24)

The pseudo-second-order model (PSO) is the most used equation, assuming chemisorption is the rate-limiting phase. The linear form describes Ni(II) biosorption with a strong correlation coefficient (R2 > 0.99)^38–44^.

Although FT-IR analysis suggested that the interaction between Ni(II) ions and PFTAC involves mainly surface functional groups through weak physical adsorption, the kinetic and thermodynamic parameters indicate that chemisorption is the dominant mechanism. The pseudo-second-order kinetics (R² = 0.9957) and positive ΔH° value confirm that chemical bonding and energy-dependent interactions occur between Ni(II) ions and the oxygen-containing groups on the carbon surface. These interactions primarily involve ion exchange and surface complexation with –OH, –COOH, and –PO₄^−3^ groups introduced during phosphoric acid activation. The H₃PO₄ treatment enhances surface acidity, increases porosity, and generates phosphate and hydroxyl groups that act as coordination sites for Ni(II) binding. Therefore, the overall adsorption process can be described as a synergistic mechanism, in which chemisorption through ion exchange and complexation predominates, supported by minor contributions from physical adsorption due to the high surface area and mesoporosity of the PFTAC material.

### Weber and Morris model

Because internal diffusion controls the adsorption rate in most liquid systems, the Weber and Morris model, also known as the intra-particle diffusion model, is highly relevant. A general illustration of kinetics can be seen in Eq. (9), where the predicted value of the exponent is 0.5, and the intercept is related to mass transfer across the boundary layer.

9 $$\:{q}_{t}=\:{K}_{m}{t}^{0.5}+C$$

where k_m_ is the intra- particle diffusion rate constant (mg/g min^1/2^).

**Fig. 25:**
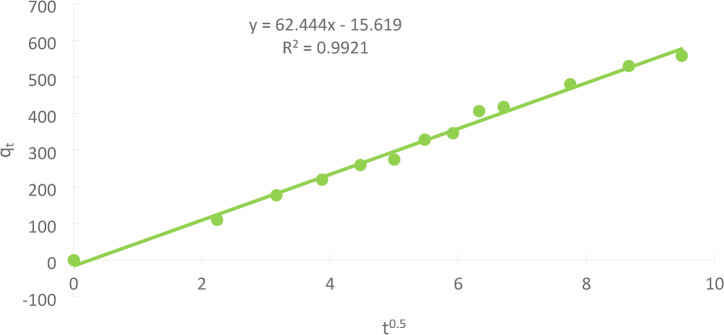
The Weber and Morris model at (initial concentration = 50 ppm, PFTAC dose = 0.5 g/250 ml, stirring speed = 300 rpm T = 301 K and time = 90 min, pH = 3).

Figure 25 depicts the plot of qt vs. t ^1/2,^ giving a straight line with a slope k_m_ and intercept C when intra-particle diffusion is engaged in adsorption. The values of C can roughly estimate the boundary layer thickness; the higher the C value, the thicker the boundary layer^20^.

The Weber and Morris intra-particle diffusion model was applied to examine the rate-controlling step of Ni(II) adsorption onto PFTAC. The plot of q_t versus t^0.5 (Fig. 25) yielded a linear relationship with a high correlation coefficient (R² = 0.9921) but a non-zero intercept (C = − 15.619 mg/g), indicating that intra-particle diffusion is not the sole rate-limiting mechanism. The negative intercept suggests that boundary layer effects also contribute significantly to the adsorption kinetics. Therefore, the adsorption process is controlled by a combination of intra-particle diffusion and external mass transfer across the boundary layer, rather than by pore diffusion alone. This observation aligns with the pseudo-second-order kinetic model (R² = 0.9957), which reflects chemisorption as the dominant mechanism, supported by both surface and intra-particle interactions.

The adsorption kinetics of Ni(II) onto PFTAC were evaluated using pseudo-first-order, pseudo-second-order, and intra-particle diffusion (Weber–Morris) models. The experimental data fit best with the pseudo-second-order model (R² = 0.9957), indicating that chemisorption likely governs the rate-limiting step. The pseudo-first-order model showed a lower correlation (R² < 0.92), suggesting that physisorption plays a minor role. The Weber–Morris intra-particle diffusion plot revealed a multilinear profile with a non-zero intercept, indicating that while internal diffusion contributes to the adsorption process, boundary layer effects are also significant. These results collectively suggest that the adsorption mechanism is predominantly controlled by chemisorption on the available active sites of PFTAC, with contributions from both surface adsorption and intra-particle diffusion. Understanding the kinetic behavior provides insights into optimizing contact time and predicting adsorption efficiency in practical applications.

From Table 2, it was possible to compute the kinetic models and other variables related to the Ni(II) adsorption onto PFTAC throughout a contact time of 90 min at 300 rpm and 25 °C.

**Table 2:** The kinetic model’s parameters for Ni(II) adsorption.

Kinetic models	Parameters	Weight of adsorbent = 1 g
Pseudo 1 st order equation	q_e_ (EXP.)(mg/g)	1235.7
	q_e_ (calc.)	557.37
	R^2^	0.3772
	K_1_(min^− 1^)	0.0983
Pseudo 2 nd order equation	q_e_ (calc.)	557. 37
	R^2^	0.9957
	K_2_ (g/mg.min)	$\:3.9\times\:{10}^{-4}$
Weber and Morris model	C	−15.619
	R^2^	0.9921
	K_m_(mg g^− 1^min^− 1^)	62.444

### Thermodynamic parameters

The standard Gibbs free energy (G°), standard enthalpy change (H°), and standard entropy change (S°) were among the crucial thermodynamic parameters that were calculated using the data from the adsorption equilibrium acquired at various temperatures. Equation (10) was used to determine Ni (II)adsorption’s standard Gibbs free energy (G°).

10 $$\:\varDelta\:{G}^{0}=-RTln{K}_{e}$$

11 $$\:{K}_{e}=\frac{{q}_{e}}{{C}_{e}}$$

11The adsorption equilibrium constant, Ke, e, was calculated using Eq. at each temperature.

Where q_e_ (mg/g) is the amount of Ni(II) adsorbed from the solution at equilibrium C_e_(mg/l), the equilibrium concentration Ni(II) in the solution, R (j/mol.k) the gas constant = 8.314, T(^˚^k) the absolute temperature.

12 $$\:\mathrm{L}\mathrm{n}\:{\mathrm{K}}_{\mathrm{e}}=\:-\left(\frac{{\varDelta\:\mathrm{H}}^{0}}{\mathrm{R}\mathrm{T}}\right)+\left(\frac{{\varDelta\:\mathrm{S}}^{0}}{\mathrm{R}}\right)$$

**Fig. 26:**
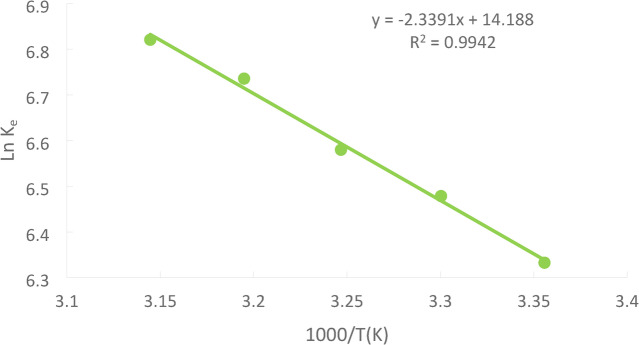
Van̕ t Hoff̓ s for the solution of initial Ni (II)concentration 50 ppm at pH = 3, PFTAC dose 0.5 g/250 ml at 300 rpm, contact time 90 min at different temperature.

∆H˚ and ΔS° were obtained from the slope and intercept, respectively, of the van̕ t Hoff̕ s plot of ln (k_eq_) versus 1/T; Eq. (12) as shown in Figure 26 and for a Ni(II).

The values of ΔG°, ∆H˚, and ΔS° in Table 3 indicate that significant energy is needed to exchange the divalent Ni(II), as indicated by the positive value of H. S°’s positive value denotes the presence of both an adsorbent and an adsorbate. A negative value for G° indicates that adsorption processes are feasible and spontaneous^6^.

**Table 3:** Thermodynamic parameters.

Nickel (II) Concentration 50 ppm Remove by PFTAC	T(K)	298	303	308	313	318
	1000/T(K^− 1^)	3.36	3.30	3.25	3.19	3.14
	q_e_	557.74	554.318	584.365	529.703	540.103
	C_e_	0.99	0.9132	0.933	0.6292	0.5892
	K_e_	562.6262	607.0061	626.329	841.8675	916.6718
	lnK_e_	6.33	6.48	6.58	6.74	6.83
	ΔG°(kj/mol)	−15689.5	−16,144	−16490.7	−17,528	−18033.1
	∆H˚(Kj/mol)	19.45				
	ΔS°(Kj/mol.K)	117.96				

### Isothermal models

Analyzing equilibrium data is essential and crucial for determining the maximum capacity of adsorbents. It also has a crucial function in determining the maximum capacity of adsorbents. Creating an equation that accurately captures the outcomes and can be applied to design is crucial. The most frequently cited equations for the equilibrium modeling of an adsorption system are the Freundlich equation and the Langmuir equation.

### Langmuir adsorption isotherm

According to the Langmuir model, no interaction between sorbed species and Ni(II) is taken up by monolayer adsorption on a homogeneous surface. The following is how the Langmuir Eq. (13) is written :

13 $$\:\frac{{\mathrm{C}}_{\mathrm{e}}}{{\mathrm{q}}_{\mathrm{e}}}=\frac{1}{{\mathrm{q}}_{\mathrm{m}\mathrm{a}\mathrm{x}}\times\:\mathrm{b}}+\frac{{\mathrm{C}}_{\mathrm{e}}}{{\mathrm{q}}_{\mathrm{m}\mathrm{a}\mathrm{x}}}$$

Where: q_e_ is the equilibrium Ni(II) concentration on the adsorbent (ppm), C_e_ is the equilibrium Ni(II) in the solution, q_max is_ the adsorbent’s monolayer adsorption saturation capacity, and b is the Langmuir constant.

**Fig. 27:**
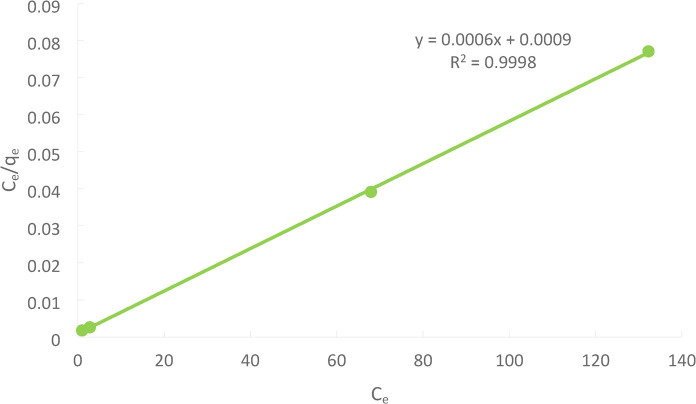
Langmuir adsorption isotherm for Ni(II) adsorption for the solution of initial Ni(II)concentration = 50 ppm at pH = 3, PFTAC dose 0.5 g/250 ml at 300 rpm, contact time 90 min at temperature = 25℃.

The linear plot in Figure 27 shows that the adsorption verifies the Langmuir isotherm, the Langmuir adsorption constant.

### Freundlich adsorption isotherm

Freundlich adsorption isotherm. One of the most widely used mathematical descriptions usually fits the experimental data over various concentrations. This isotherm expresses surface heterogeneity and an exponential distribution of active sites and their energies. The non-linear form of the Freundlich model is expressed as :

14 $$\:{\mathrm{q}}_{\mathrm{e}}={\mathrm{K}}_{\mathrm{f}}\:{\left({\mathrm{C}}_{\mathrm{e}}\right)}^{\raisebox{1ex}{$1$}\!\left/\:\!\raisebox{-1ex}{$\mathrm{n}$}\right.}$$

The linear form of the Freundlich model is expressed as:

15 $$\:\mathrm{log}{q}_{e}=\mathrm{log}{K}_{f}+\left(\frac{1}{n}\right)\mathrm{log}{C}_{e}$$

**Fig. 28:**
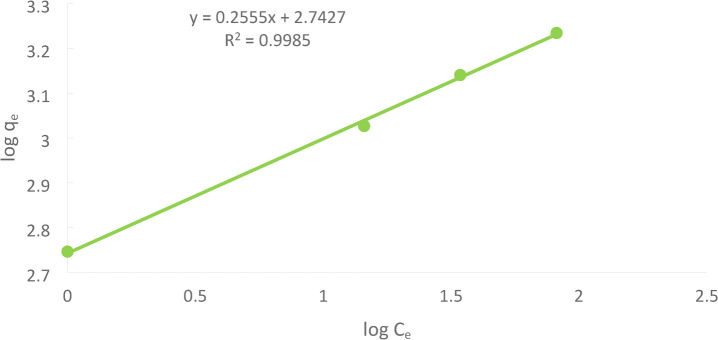
Freundlich Ni(II) adsorption for the solution of initial Ni(II)concentration = 50 ppm at pH = 3, PFTAC dose 0.5 g/250 ml at 300 rpm, contact time 90 min at temperature = 25℃.

Where K_F_ is Freundlich constant representing adsorption capacity and n is a constant related to the sorption intensity, which varies with the heterogeneity of the adsorbents. A plot of log q_e_ versus log C_e_ gives a straight line with a slope(1/n) and intercept (log K_F_), as shown in Figure 28.

The linear regression analysis for the isotherm models revealed that Langmuir was the best model for describing heavy metal adsorption from tannery effluents using ACTPF adsorbents. The sequence of best fit for two-parameter isotherm models is Langmuir, Freundlich, as presented in Table 4. Similar findings were also obtained from earlier studies^45^. –^46^.

The maximum adsorption capacity (qₘ) achieved with the H₃PO₄-treated palm-frond activated carbon (PFTAC) for Ni(II) ions—166.7 mg/g is notably high when placed in the context of other biomass-derived activated carbons. For example, certain studies on palm-kernel shell derived carbons and other agro-waste precursors report much lower qₘ values, indicating that PFTAC stands out for its enhanced uptake potential. In this regard, the superior performance of PFTAC underscores the effectiveness of both the palm-frond precursor and the chemical activation via phosphoric acid in creating a highly adsorptive material.

Moreover, when benchmarked against commercial activated carbons or other conventional precursors, the high qₘ value of 166.7 mg/g signifies a meaningful advancement in adsorbent development. The chemical activation method applied in our work not only increases surface area and porosity, but also introduces functional groups that enhance metal-ion binding. Consequently, PFTAC’s performance puts it in a strong position for practical industrial wastewater applications, especially when cost and sustainability of the adsorbent are taken into account^47,48^.

**Table 4:** Shows a comparison between Langmuir and Freundlich models.

	Langmuir isotherm constants			Freundlich isotherm constants		
	q_max_(mg/g)	b (L/mol)	*R* ^2^	1/*n*	K_f_(mg/g)	*R* ^2^
Nickel (II)	166.7	1.5	0.9998	0.255	553	0.9985

### RSM analysis for Ni(II) percent removal (%) optimization

RSM examined the adsorption processes based on the Box-Behnken design (BBD) in a standard manner. Initial studies with a single parameter were carried out to determine the important factors affecting the response, for instance, time(min), concentration(ppm), and temperature(ºc) that affected Ni(II) percent removal (%) using PFTAC. Several experiments were carried out after selecting the parameter levels shown in Table 5. Table 5 summarises the results of the statistical plan and response operations.

**Table 5:** BB factorial design of Ni(II) percent removal (%) optimization using PFTAC.

Coded values response (%)								
**Trial**	**X**_**1**_	**X**_**2**_	**X**_**3**_	**Time** **(min)**	**Concentration** **(ppm)**	**Temperature(ºc)**	**Ni(II)percent removal (%) using** PFTAC	
							**Measured**	**Predicted**
1	0	−1	1	30	50	45	64.57	58.8894
2	0	0	0	30	150	35	54.02	52.9254
3	1	0	1	90	150	45	82.92	83.0134
4	0	0	0	30	150	35	54.02	52.9254
5	1	−1	0	90	50	35	98.43	100.6894
6	0	−1	−1	30	50	25	44.42	47.4814
7	1	0	−1	90	150	25	82.25	77.5654
8	−1	1	0	0	300	35	0	0
9	0	0	0	30	150	35	54.02	52.9254
10	−1	−1	0	0	50	35	0	0
11	1	1	0	90	300	35	46.65	42.6144
12	0	1	−1	30	300	25	27.89	27.9064
13	0	1	1	30	300	45	37.19	31.3144
14	−1	0	1	0	150	45	0	0
15	−1	0	−1	0	150	25	0	0

The results illustrated that the Ni(II)percent removal (%) Different values of the three primary components utilizing PFTAC were different. Because of the outcomes of the study and Table 5, a small difference between the measured and predicted Ni(II)percent removal (%) using PFTAC nominated the high precision of the model in estimating the response variable (Ni(II)percent removal (%))^47^. For that reason, adding to the experimental result, the expected results are also illuminated using the response surface methodology, i.e.(Box-Behnken design), which is used for the optimization of Ni(II)percent removal (%) using PFTAC^50^.

ANOVA and the F test were used to evaluate a model’s efficacy, with the findings in Table 6^52^.

The following equation perfectly fits the quadratic model’s description of the statistical relationship between the chosen components and the response variable (Ni(II)percent elimination (%)) in terms of coded factors.

$$\:Y\left(\%\right)=-68.8481+2.4584{x}_{1}+0.1707{x}_{2}+2.9384{x}_{3}-0.0127{x}_{1}^{2}-0.0004{x}_{2}^{3}-0.0317{x}_{3}^{2}-0.0023{x}_{1}{x}_{2}-0.0023{x}_{1}{x}_{3}-0.0016{x}_{2}{x}_{3}\to\:16$$

In the above equations(16), X_1_, X_2,_ and X_3_ were the linear parameters; X_1_^2^, X_2_^2^, and X_3_^2^ were second-order parameters of the model, in conjunction with the interaction parameters X_1_ × _2_, X_1_ × _3,_ and X_2_ × _3_^51^.

Also, Y is the response (Ni(II)percent removal (%)), and X_1_, X_2,_ and X_3_ are the coded values of the test variables, i.e., Time(min), concentration(ppm), and Temp. (ᵒc), respectively.

**Table 6:** ANOVA results for Ni(II)percent removal (%)using PFTAC.

Source	Sum of Square	df	Mean of Square	F-Value	*p*-Value	Significant or insignificant model terms
Model	11951.64	3	3983.879	15.88996	0.000260	Significant

The standard deviation of the data from the mean value is the parameter F-value. The model predicts test results when the F-value is very high, and the “prob> F” value is less than 0.05. A 95% confidence level statistical analysis shows that it is significant. Values greater than 0.1000 indicate that the model terms are not important. Table 6 shows that this model’s F-value and p-value are high (15.88996) and less than 0.05 (15.88996), respectively, indicating it was fully significant. ^(52–55)^

According to this model’s RSM approach, values of R^2^ and R^2^_adj_ that are close to 1 (R^2^ of 0.991 and R^2^_adj_of 0.974) show a stronger correlation between experimental and theoretical values^56^.

**Fig. 29:**
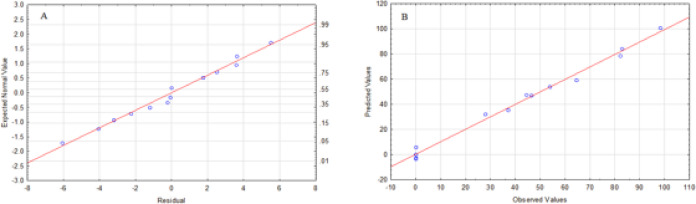
Distributive plotting of A)Normal plot of residuals, B) predicted values Vs. observed values for Ni(II)percent removal (%)using PFTAC).

Explains the viability of a model dependent on several assumptions, such as residuals, constant variance (∂2), and independent residuals Figure 29 A depicts the expected normal value plot of the residual for Ni(II)percent elimination (%)with PFTAC. Since all plots are linear and there were no deviations in the residuals’ normality, the residuals tail a normal distribution^57^.

Figure 29 B shows that the data points on the plot have a linear distribution, which supports a strong connection between the predicted and experimental values. Using PFTAC, remove % of Ni(II),

**Fig. 30:**
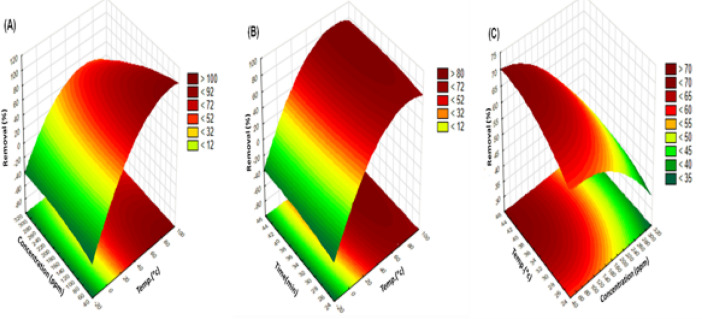
Response surface plots for Ni(II)percent removal (%)using PFTAC A)effect of Concentration/Temp. [at Dose: 0.5gm; at time:90 min]; B)effect of Time/Temp [at Ni(II) concentration = 50 ppm; at Dose: 0.5gm]; C)effect of Temp/Concentration at[time:90 min; at Dose: 0.5gm].

In 3D presentations with contours, the Box-Behnken design’s findings can be displayed. The 3D surface plots show the interactions between the parameters examined and allow for the creation of ideal conditions. Figure 30 presents these graphs of the second-order polynomial equation for Ni (II) elimination percent (%) utilizing PFTAC with two variables remaining constant and two additional variables falling within the predetermined experimental ranges. For the pair-wise combination of the three factors (X_1_ × _2_, X_1_ × _3_, X_2_ × _3_), the 3D plots of the response surface and its 2D contour plot were created while retaining the third factor at its central point level (0).

The effect of concentration/temperature on Ni(II)removal (%) using PFTAC is shown in Fig. 30A. Maximum Ni (II) removal (%) was at 50 ppm, and Ni (II) removal (%) decreased as concentration increased. The uptake rate is regulated by the rate at which the adsorbate is transferred from the outer to the inner sites of the adsorbent particles. This may be because there is more surface area of the adsorbent available initially for the adsorption of Ni (II) ions, but after Ni (II) concentration increases, the surface adsorption sites become exhausted.

As opposed to this, Fig. 30B illustrates the effect of Time/Temp, Ni (II) removal percent (%) using PFTAC, which rose with an increase in time from 0 to 90 min because the networks of the adsorbent spread quickly at first and reached equilibrium after that time. As a result, for the first time, MB molecules found it considerably simpler to enter PFTAC’s interior and mix with the adsorption sites^58^.

Figure 30C for PFTAC additionally displays the fitted surface plotting the percent Ni (II) removal against the combined effects of temperature and concentration. However, by examining the combined effects of temperature and concentration on the percentage of Ni (II) removed, it was recognized that the percentage of Ni (II) removed increases when the temperature is high, and the concentration is low. Additionally, as the temperature climbed from 25 to 45 °C, the Ni(II) removal (%) increased. This can be explained by the fact that when the temperature increased, the adsorbate molecule diffusion rate across the adsorbent particles’ interior pores and the external boundary layer reduced, and the desorption efficiency increased^53,58^.

## Conclusion

This study successfully demonstrated the effectiveness of activated carbon derived from palm fronds and treated with H₃PO₄ (PFTAC) for the adsorption of Ni(II) ions from aqueous and industrial wastewater. The adsorbent exhibited excellent performance, achieving a maximum removal efficiency of 99.65% within 90 min at an initial Ni(II) concentration of 50 ppm under neutral pH conditions. Characterization analyses (SEM, FT-IR, and XRD) confirmed the porous, functionalized, and partially crystalline nature of PFTAC, while BET, t-plot, and BJH analyses verified its mesoporous structure and high surface area. Adsorption kinetics followed the pseudo-second-order model (R² = 0.9957), indicating that chemisorption was the rate-limiting step. The Langmuir isotherm model provided the best fit (R² = 0.9998) with a maximum adsorption capacity of 166.7 mg/g, suggesting monolayer adsorption on a homogeneous surface. Thermodynamic parameters indicated that the process was spontaneous and endothermic. Optimization using the Box–Behnken Design (R² = 0.991, *p* < 0.05) confirmed that contact time, initial concentration, and temperature were the most significant factors influencing adsorption efficiency.Moreover, the regenerated PFTAC maintained high adsorption efficiency over multiple reuse cycles, confirming its structural stability and cost-effectiveness for real industrial wastewater treatment. Therefore, H₃PO₄-treated palm frond activated carbon can be considered a sustainable, eco-friendly, and highly efficient adsorbent for large-scale Ni(II) removal applications.Future work should focus on evaluating the adsorption performance of PFTAC in continuous flow systems, assessing its regeneration under real industrial conditions, and exploring its potential for removing multiple heavy metals simultaneously to enhance its practical applicability in wastewater treatment plants.

## Data Availability

All data generated or analysed during this study are included in this published article.
